# Post-endoscopic Retrograde Cholangiopancreatography Complications: A Case of Duodenal Perforation and Literature Review

**DOI:** 10.7759/cureus.40303

**Published:** 2023-06-12

**Authors:** Rabira R Dufera, Tamiru B Berake, Benedict Maliakkal

**Affiliations:** 1 Internal Medicine, Meharry Medical College, Nashville, USA; 2 Internal Medicine, Tufts Medical Center, Boston, USA; 3 Gastroenterology and Hepatology, Nashville General Hospital, Nashville, USA

**Keywords:** post-ercp pancreatitis, ercp complications, perforated duodenum, cholangitis and endoscopic retrograde, endoscopic retrograde cholangio-pancreatography, endoscopic perforation, post-ercp, post-ercp cholecystitis, diagnostic and therapeutic ercp

## Abstract

A duodenal perforation is a serious complication that can occur during endoscopic retrograde cholangiopancreatography (ERCP), particularly if it is associated with therapeutic endoscopic sphincterotomy. Therefore, it is crucial to identify and manage it early to achieve the best possible outcome. Conservative management may be attempted; however, surgical intervention is required if signs of sepsis or peritonitis are present. In this case report, we present the case of post-ERCP duodenal perforation in a 33-year-old female with sickle cell disease who presented on account of abdominal pain. The patient was diagnosed with post-ERCP duodenal perforation, type 4 according to the Stapfer classification. She was subsequently treated conservatively with intravenous antibiotics, bowel rest, and serial abdominal exams. The patient noted significant interval improvement in symptoms and was subsequently discharged home. The early detection and management of suspected complications of ERCP provide a critical prognostic value.

## Introduction

Endoscopic retrograde cholangiopancreatography (ERCP) is an advanced endoscopic procedure used for both diagnostic and therapeutic purposes. It is an invasive procedure that helps diagnose and treat pancreatic and biliary diseases. However, due to its complexity, the risk of complications like duodenal perforation has increased. In about 5%-10% of cases, adverse events can occur during the procedure [1]. These adverse events can include post-ERCP pancreatitis, perforation, infections such as cholangitis, cholecystitis, bleeding, and sedation-related side effects. To prevent these adverse events, it is crucial to select patients based on appropriate indications. Once it is decided that ERCP is necessary, mitigation efforts such as prophylactic pancreatic stent placement and rectal indomethacin should be undertaken if the patient or procedural factors suggest an increased risk of post-ERCP pancreatitis [2]. Compared with other procedures, colonoscopy, one of the most performed procedures, has a 0.1% perforation and a 1%-4% post-polypectomy bleeding rate. On the other hand, ERCPs have a 0.34% rate of perforation and a 2% post-sphincterotomy bleeding rate [3]. ERCP is now considered more of a therapeutic option than a diagnostic modality, except for patients with potential sphincter of Oddi dysfunction (SOD) due to a higher risk of complications.

## Case presentation

A 33-year-old female with a history of sickle cell disease, cholelithiasis, and choledocholithiasis presented to the emergency department with abdominal pain one day post-ERCP for choledocholithiasis. The pain was mid-abdominal radiating to the right side of her back. It was crampy, 8/10 in severity, worsened by lying flat, and alleviated by lying on her right. The patient also reported nausea, but denied any vomiting; she also had bowel movement a day prior to the presentation. There was no abdominal distension nor any urinary frequency, urgency, dysuria, fever, or chills. She also denied any chest pain, cough, joint pains, headache, or blurring of vision. She did not have any prior history of abdominal or pelvic surgeries. Her last sickle cell pain crisis was one year prior to her current hospital admission. On admission, her vitals were within normal limits. Her abdominal examination was remarkable for mild subcutaneous emphysema on the right flank, active bowel sounds, and soft abdomen with mild tenderness around the mid-abdomen. No rebound tenderness or rigidity of the abdomen was noted. She also had splenomegaly that was present at her baseline.

**Figure 1:**
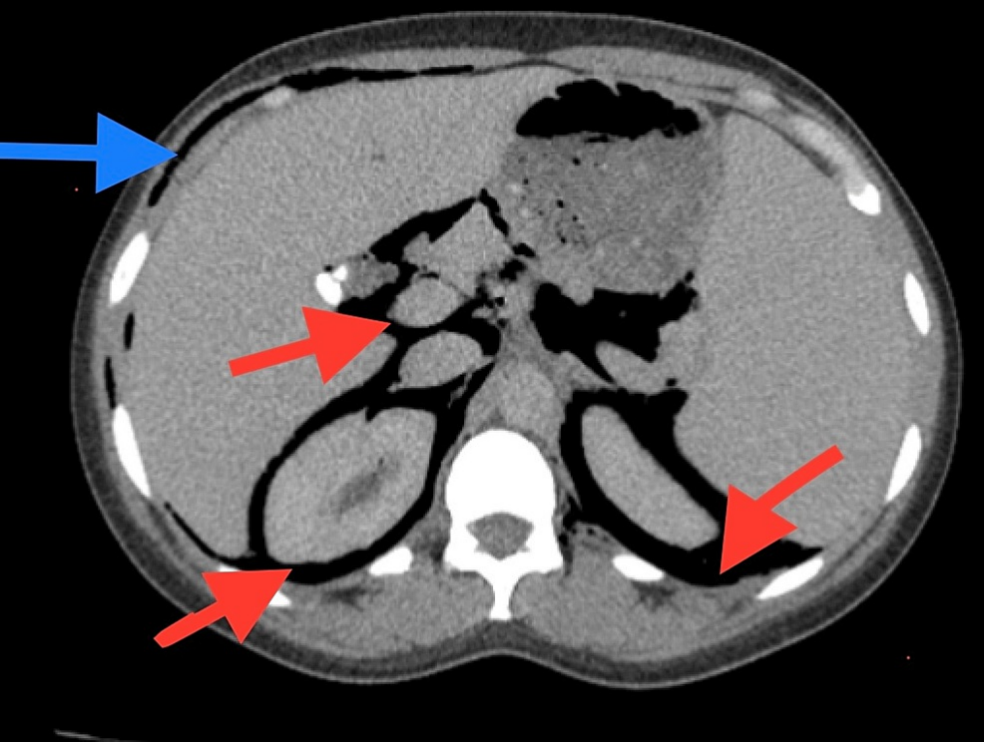
Computed tomography of the abdomen/pelvis with intravenous contrast showing subcutaneous emphysema (blue arrow) and pneumoperitoneum/retroperitoneum (red arrows)

Labs were notable for low hemoglobin at 9.6 g/dl (reference, 12.0-16.0 g/dl), unremarkable urine analysis, and unremarkable white/red blood cell count as well as platelets. The reticulocyte percentage was high at 8.14% (reference, 0.5%-1.8%). Total bilirubin was elevated at 3.0 mg/dl (reference, 0.2-1.3 mg/dl), but direct bilirubin, amylase, and lipase levels were normal. Serum sodium, potassium, chloride, blood urea, serum creatinine, alkaline phosphatase, and alanine/aspartate aminotransferase levels were also normal. Computed tomography (CT) of the abdomen/pelvis was done with intravenous contrast revealing pneumoperitoneum, pneumo-retroperitoneum, subcutaneous emphysema with splenomegaly as well as mild ascites (Figure 1).

**Figure 2:**
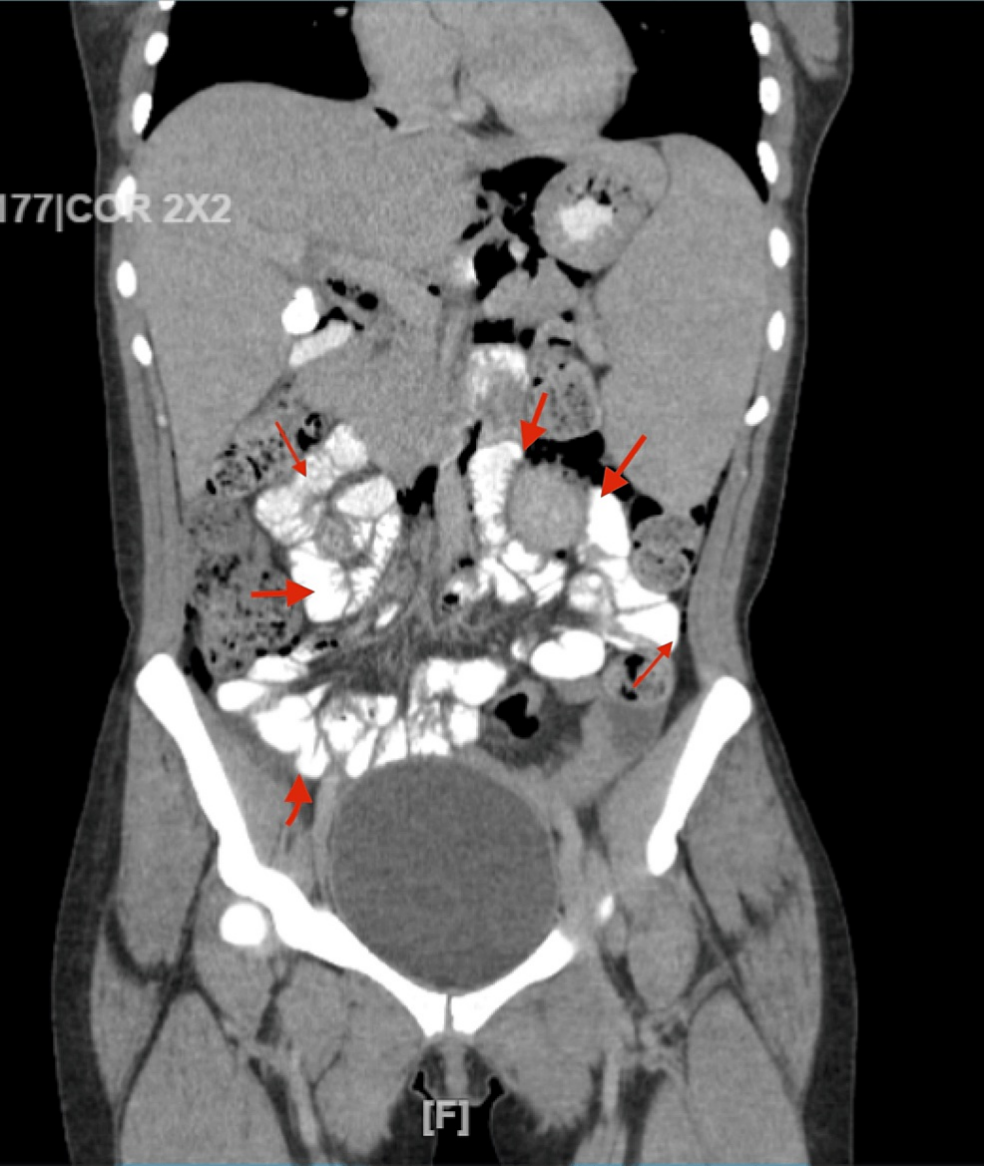
CT of the abdomen with oral contrast showing no intraperitoneal or retroperitonal contrast extravasation. The red arrows show contrast within the stomach, duodenum as well as bowels.

A conservative management plan was chosen after consultation with the surgeon. The patient was started on lactated Ringer's solution, and was kept nil per os (NPO) except oral medications. The patient was also put on intravenous (IV) antibiotics with ciprofloxacin 400 mg twice a day, and metronidazole 500 mg IV three times a day. A nasogastric tube was placed, and analgesics were administered on an as needed basis to manage her pain. She was monitored with serial vital sign checks and serial abdominal exams. Also, CT of the abdomen with oral contrast was done that did not show any contrast leak (Figure 2).

Patient's condition remained stable with no signs of infection or abdominal pain observed in the following days after her ERCP. On the third day of her admission to the hospital, she began eating and her diet was gradually advanced to regular diet the next day. She was discharged on the fifth day post-ERCP in excellent overall health with her subcutaneous emphysema and abdominal pain fully resolved. The patient followed up with her surgeon and gastroenterologist two weeks after her discharge and returned to regular check-ups. However, the patient refused to undergo laparoscopic cholecystectomy with common bile duct exploration.

## Discussion

ERCP is a procedure that uses an endoscope for diagnostic and therapeutic purposes, treating a variety of pancreaticobiliary disorders, such as bile duct stones, biliary obstruction, acute cholangitis, and post-surgery complications. However, due to the high risks involved, specialized training and experience are required. Post-ERCP pancreatitis is the most frequent complication, but post-ERCP pain can also result from acute pancreatitis and duodenal perforation [4]. Additional complications include infections like cholangitis, as well as injuries to the esophagus, liver, and spleen. Complete drainage of the obstructed biliary system during ERCP is critical in reducing the risk of post-ERCP cholangitis.

Duodenal perforation

During ERCP, the chance of perforation is generally low, at less than 1% [5]. However, there are some risk factors that can increase the likelihood of duodenal perforation. These include a deep incision outside the papilla, precut papillotomy, guidewire perforation, non-dilated bile ducts, sphincter of Oddi dysfunction, Billroth II procedure, old age, dilated bile duct, sphincterotomy, and a longer procedure duration [6]. Duodenal injuries can be classified into four types based on the location, severity, and mechanism of injury according to Stapfer et al. [7]. There are other classification systems of post-ERCP duodenal perforations by different scholars including the one by Howard et al., which divides them into type I perforations (mainly due to the guidewire), type II periampullary perforations, and type III duodenal wall perforations [8].

For the sake of our discussion, we will use the Stapfer et al. classification system. Accordingly, type 1 perforations, also known as medial or lateral wall perforations, affect the luminal wall of the duodenum and can result in large contrast leaks in the retroperitoneal or intraperitoneal space. These perforations are typically caused by too much pressure applied to the sweep of the thin-walled duodenum or when the scope tip directly perforates a duodenal diverticulum. Type 2 perforations are caused by sphincterotomy or precut needle-knife and are located in the peri-ampullary region of the duodenum. These perforations require surgical repair, but there have been successful attempts to close them endoscopically using self-expandable metallic stent(s) (SEMS). Type 3 perforations occur during cannulation of the bile and pancreatic duct and often involve guidewire perforations through a side branch of the pancreatic duct or hepatic capsule. They are usually small and related to wire or basket instrumentation near an obstructing entity. Finally, type 4 perforations are retroperitoneal micro-perforations that are not considered true perforations. They are characterized by retroperitoneal air alone and are probably related to the use of compressed air to maintain clearance of the lumen [8].

Clinical presentation of duodenal perforations

Post-ERCP duodenal perforation can have a range of clinical symptoms, from mild to severe. These can include abdominal pain, subcutaneous emphysema, peritonitis, fever, cold sweating, and vomiting. Type 1 perforations are usually identified immediately, while type 2 can be identified through radiologic evidence of air in the retroperitoneum or contrast extravasation during ERCP or CT evaluation for post-ERCP pain. Type 3 perforations occur during cannulation of the bile and pancreatic duct and involve guidewire perforations. The patient in this case presentation had a type 4 perforation [9].

Diagnosis

In the case of a suspected perforation in a patient, an abdominal CT scan is highly recommended. It is the most effective method to detect and pinpoint the location of the perforation. Although a diagnosis can be made or suspected during ERCP, it is important to note that only 27% of duodenal perforations related to ERCP are detected during the procedure itself [10]. To confirm a suspected perforation during ERCP, gastrograffin can be used for upper gastrointestinal imaging to confirm the perforation through contrast extravasation. Additionally, a chest X-ray can also reveal air under the diaphragm, subcutaneous emphysema, pneumomediastinum, or even tension pneumothorax, making it another diagnostic method to consider.

Management

The management of complications arising from ERCP procedures can be greatly improved by avoiding unnecessary procedures in the first place. If ERCP is deemed necessary, it is important to communicate the potential benefits and risks of the procedure to the patient and their family. The experience of the endoscopist is a key factor in reducing the likelihood of complications. However, if complications do arise, prompt identification and management are crucial. Type 1 perforations can be treated through endoscopic methods such as using clips, an over-the-scope clip device, or an endoscopic suturing device. However, if there is significant contrast extravasation, surgery is typically necessary in most cases [10-12]. Surgical options include choledochotomy with stone extraction and T-tube drainage, repair of the perforation, drainage of an abscess or phlegmon, choledochojejunostomy, or pancreatoduodenectomy.

Type 2 perforations can be subtle and easily missed, but careful assessment of fluoroscopy during ERCP, especially the gas patterns, can help avoid delays in treatment. If there is still some uncertainty about the presence of the perforation, computed tomography can be used for confirmation. Type 2 perforations that are identified during ERCP can be closed using through-the-scope clips (TTSCs) or a fully covered SEMS [10,11]. Type 3 perforations are usually managed conservatively with intra-ductal stent placement and antibiotics [11]. Surgery is indicated for type 2 and type 3 perforations only when there is excess free intraperitoneal or retroperitoneal fluid, retained stones, hardware, or unrelieved bile obstruction.

Type 4 perforations may not require additional treatment or workup if the abdominal examination is normal or if there is no evidence of contrast extravasation. In cases where minimal contrast extravasation is detected, upper gastrointestinal imaging should be done within eight hours to confirm the presence of a leak. Double-contrast CT is necessary to check for the development of intraperitoneal or retroperitoneal fluid accumulations. These studies should be repeated regularly until clinical recovery appears certain [11,12].

## Conclusions

Complications following an ERCP procedure are not uncommon. Therefore, endoscopists must have a clear justification for performing ERCP and prioritize therapeutic purposes over diagnostic indications as there are less invasive diagnostic modality methods available. Patients must be duly informed about the potentially severe complications that may arise after the procedure. It is imperative to remain vigilant and well-prepared to perform necessary procedures, such as placing pancreatic stents, to prevent high-risk post-ERCP pancreatitis. Alternatively, pharmacological methods can be utilized for prevention. The early detection and appropriate management of complications can avert serious consequences, including fatal ones in a synopsis.

## References

[1] Rustagi T, Jamidar PA (2015). Endoscopic retrograde cholangiopancreatography-related adverse events: general overview. Gastrointest Endosc Clin N Am.

[2] Balmadrid B, Kozarek R (2013). Prevention and management of adverse events of endoscopic retrograde cholangiopancreatography. Gastrointest Endosc Clin N Am.

[3] Enns R, Eloubeidi MA, Mergener K, Jowell PS, Branch MS, Pappas TM, Baillie J (2002). ERCP-related perforations: risk factors and management. Endoscopy.

[4] Cooper ST, Slivka A (2007). Incidence, risk factors, and prevention of post-ERCP pancreatitis. Gastroenterol Clin North Am.

[5] Andriulli A, Loperfido S, Napolitano G (2007). Incidence rates of post-ERCP complications: a systematic survey of prospective studies. Am J Gastroenterol.

[6] Cotton PB, Garrow DA, Gallagher J, Romagnuolo J (2009). Risk factors for complications after ERCP: a multivariate analysis of 11,497 procedures over 12 years. Gastrointest Endosc.

[7] Stapfer M, Selby RR, Stain SC, Katkhouda N, Parekh D, Jabbour N, Garry D (2000). Management of duodenal perforation after endoscopic retrograde cholangiopancreatography and sphincterotomy. Ann Surg.

[8] Howard TJ, Tan T, Lehman GA (1999). Classification and management of perforations complicating endoscopic sphincterotomy. Surgery.

[9] Silviera ML, Seamon MJ, Porshinsky B (2009). Complications related to endoscopic retrograde cholangiopancreatography: a comprehensive clinical review. J Gastrointestin Liver Dis.

[10] Kumbhari V, Sinha A, Reddy A (2016). Algorithm for the management of ERCP-related perforations. Gastrointest Endosc.

[11] Vezakis A, Fragulidis G, Nastos C, Yiallourou A, Polydorou A, Voros D (2011). Closure of a persistent sphincterotomy-related duodenal perforation by placement of a covered self-expandable metallic biliary stent. World J Gastroenterol.

[12] Lee TH, Han JH, Park SH (2013). Endoscopic treatments of endoscopic retrograde cholangiopancreatography-related duodenal perforations. Clin Endosc.

